# Outbreak of *Salmonella* Typhimurium Infections Linked to Commercially Distributed Raw Milk — California and Four Other States, September 2023–March 2024

**DOI:** 10.15585/mmwr.mm7427a1

**Published:** 2025-07-24

**Authors:** Eva Weinstein, Katherine Lamba, Christian Bond, Vi Peralta, Michael Needham, Stephen Beam, Francine Arroyo, David Kiang, Yishi Chen, Seema Shah, Mark E. Beatty, Stephen Klish, Akiko Kimura

**Affiliations:** ^1^California Department of Public Health; ^2^California Department of Food and Agriculture; ^3^County of San Diego Health and Human Services Agency, San Diego, California; ^4^Orange County Health Care Agency, Anaheim, California.

**Summary**

**What is already known about this topic?**

Unpasteurized (raw) milk has been linked to foodborne illness outbreaks caused by *Escherichia coli* bacteria and certain species of *Brucella*, *Campylobacter*, *Cryptosporidium*, and *Salmonella*.

**What is added by this report?**

During October 2023–March 2024, California public health officials investigated an outbreak of *Salmonella* Typhimurium infections linked to raw milk from a California dairy farm. Among 171 cases identified in California and four other states, 70% were among children and adolescents aged <18 years. Whole-genome sequencing detected the *S*. Typhimurium outbreak strain in raw milk and raw milk cheese aged for 60 days, both produced by the dairy.

**What are the implications for public health practice?**

Commercially distributed raw dairy products have the potential to cause large and widespread infectious disease outbreaks. Public health messaging should explain the risks associated with these products to consumers, especially those at risk for severe disease, including children.

## Abstract

Unpasteurized (raw) milk has been linked to foodborne illness outbreaks caused by *Escherichia coli* bacteria and certain species of *Brucella*, *Campylobacter*, *Cryptosporidium*, and *Salmonella*. In October 2023, the County of San Diego Health and Human Services Agency notified the California Department of Public Health (CDPH) of eight cases of salmonellosis in persons who reported consuming brand A raw milk, produced exclusively by dairy farm A. A total of 171 outbreak-associated *Salmonella* Typhimurium cases were identified through review of standardized salmonellosis case report forms and a search of PulseNet, CDC’s national molecular subtyping network for enteric disease surveillance, followed by administration of a dairy-focused supplementary questionnaire. Most cases (98%) were identified in California; one case each was identified in four other states. Among the 171 cases, 120 (70%) cases and 18 (82%) of the cases requiring hospitalization were among children and adolescents aged <18 years. Among 159 patients confirmed to be infected with the outbreak strain, 55 (70%) of those with exposure data consumed brand A raw milk or heavy cream. Four of 40 samples collected from dairy farm A, retail stores, and patients’ homes, including raw milk and raw milk cheese aged for 60 days, tested positive for the outbreak strain of *S.* Typhimurium by whole-genome sequencing. Dairy farm A voluntarily recalled raw milk and raw heavy cream 1 week after the initial outbreak identification. Commercially distributed raw dairy products have the potential to cause large and widespread infectious disease outbreaks. Public health authorities should continue to raise awareness of the risks associated with consuming raw dairy products, especially by persons at increased risk for severe disease from enteric pathogens, including children.

## Introduction

In California, unpasteurized (raw) milk is regulated by the California Department of Food and Agriculture (CDFA). CDFA requires raw milk dairy farms to hold a permit and pass dairy farm and bottling sanitation inspections. Livestock must be tested for brucellosis and tuberculosis annually. Raw milk must meet strict bacterial and cell count limits and be kept at 45°F (7.2°C) or below (*1*). Raw milk may be legally sold at retail stores but requires warning labels alerting customers of potential contamination by disease-causing microorganisms (*1*). The California Code of Regulations mandates that health care providers and laboratories report *Salmonella* infections to local public health departments (LHDs) within 1 working day of identification.* LHDs attempt follow-up of reported cases by interviewing patients using a standard form that includes questions about various potential source exposures. On October 18, 2023, the County of San Diego Health and Human Services Agency notified the California Department of Public Health (CDPH) of eight cases of salmonellosis with onset dates during September 21–October 12, 2023, in persons who reported having consumed brand A raw milk. Brand A raw milk is produced exclusively by dairy farm A and is commercially distributed throughout California. This notification, coupled with a recent report from another California LHD of a patient with *S.* Typhimurium infection who consumed brand A raw milk before illness onset on October 3, 2023, prompted a statewide investigation. 

## Investigation and Findings

### Case Identification

A confirmed outbreak-associated case was defined as a laboratory-confirmed infection with *S.* Typhimurium with allele code SALM1.0 – 6745.4.2.1x that is closely related based on whole-genome sequencing (WGS) to other isolates in the same outbreak,^†^ in a person with symptom onset (diarrhea, fever, vomiting, or abdominal pain) during September 15, 2023–May 4, 2024. This period covered approximately 1 week before the first known illness onset through the end of the outbreak monitoring period, determined by the last known onset date plus additional lag time in days to account for delays in WGS and identification of confirmed outbreak-associated cases. A probable case was defined as laboratory-confirmed *Salmonella* infection without serotype and WGS results and with symptom onset during September 15, 2023–May 4, 2024 in a person who reported consuming brand A raw milk within 7 days before symptom onset. CDPH sought to identify outbreak-associated cases through review of California standardized salmonellosis case report forms for raw dairy product exposure and by searching PulseNet,^§^ CDC’s national molecular subtyping network for enteric disease surveillance, for clinical isolates related to the outbreak strain by WGS. Cases outside California were confirmed by WGS and identified in PulseNet.

CDPH requested, via email, that California LHDs and states with cases identified via the process described above reinterview patients using a supplementary questionnaire in paper form or in the REDCap electronic survey hosted at CDPH (*2*,*3*). The supplementary questionnaire contained questions about raw dairy product exposures, including the type of product, brand, purchase location and date, availability of leftover product for testing, and other exposure details.

The percentage of patients with confirmed cases who reported exposure to raw dairy products was compared with the background percentage of persons who reported raw dairy product exposure in the general population of the California catchment area included in the 2018–2019 Foodborne Diseases Active Surveillance Network Population Survey (*4*), using the binomial probability model; p-values <0.05 were considered statistically significant. CDPH deemed this activity routine public health response and not research, and it therefore did not require institutional review board review.

### Patient Demographic and Clinical Characteristics

The investigation identified 171 salmonellosis cases in California and four other states, including 159 (93%) confirmed and 12 (7%) probable cases (Figure). Known illness onset dates ranged from September 21, 2023, through March 11, 2024; 140 (82%) cases occurred during September and October 2023 (median onset date was October 11, 2023). The distribution of the cases was consistent with a continuous common source outbreak (*5*).

**FIGURE:**
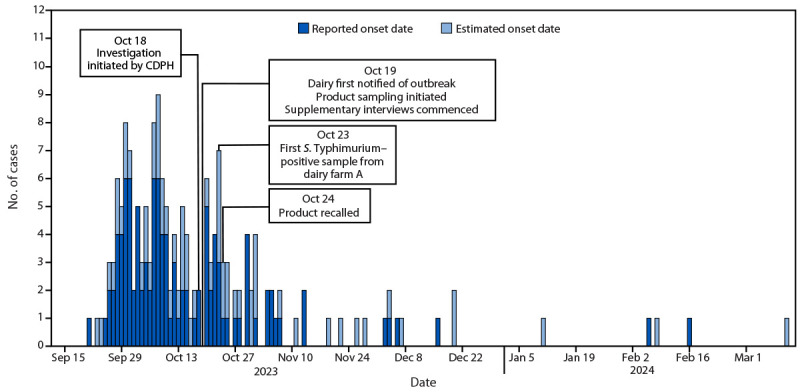
Outbreak of *Salmonella* Typhimurium linked to consumption of raw milk products, by reported* or estimated^†^ illness onset date (N = 171) — California^§^ and four other states,^¶^ September 2023–March 2024 **Abbreviation:** CDPH = California Department of Public Health. * Onset of diarrhea. ^†^ If diarrhea onset date was unavailable, symptom onset date was used; for cases missing onset date, specimen collection date minus 3 days was used. ^§^ Cases reported from 35 of 61 California local health departments. ^¶^ New Mexico, Pennsylvania, Texas, and Washington each reported a single case.

Cases were identified from five states: 167 (98%) occurred across California, in 35 of California’s 61 local health jurisdictions, and one case each was reported from New Mexico, Pennsylvania, Texas, and Washington (Table). The median patient age was 7 years (range = 9 months–87 years); 67 (39%) cases occurred in children aged <5 years, 40 (23%) in children aged 5–12 years, and 13 (8%) in adolescents aged 13–17 years. Overall, 108 (63%) patients were male, and among 136 (80%) with information on race reported, 105 (77%) were White. Among 162 patients with hospitalization information available, 22 (14%) were hospitalized, including 18 (82%) who were aged <18 years. No deaths were reported.

**TABLE:** Characteristics of patients associated with a *Salmonella* Typhimurium outbreak linked to brand A raw milk — California and four other states,* September 2023–March 2024

Characteristic (no. of cases with available information)	No. (%)^†^
**Case classification (N = 171)**	
Confirmed^§^	159 (93)
Probable^¶^	12 (7)
**State (N = 171)**	
California	167 (98)
Other*	4 (2)
**Median age (range)**	7 yrs (9 mos–87 yrs)
**Age group, yrs (N = 171)**	
<5	67 (39)
5–12	40 (23)
13–17	13 (8)
18–64	45 (26)
≥65	6 (4)
**Male sex**	108 (63)
**Hispanic or Latino ethnicity (n = 130)**	19 (15)
**Race (n = 136)**	
Asian	10 (7)
Black or African American	5 (4)
White	105 (77)
Other	16 (12)
**Hospitalized (n = 162)**	
No	140 (86)
Yes	22 (14)
**Death**	0 (—)
Raw dairy exposure information (n = 91)**	
**Any raw milk or heavy cream consumption (n = 91)**	72 (79)
**Brand (n = 72)**	
Brand A	67 (93)
Other	3 (4)
Unknown	2 (3)
**Frequency of consumption of brand A dairy products (n = 49)**	
Once only	8 (16)
Weekly	17 (35)
Daily	24 (49)
**Brand A product type (n = 68)**	
Milk only	61 (90)
Heavy cream only	1 (1)
Raw cheese only	1 (1)
More than one product^††^	5 (7)

* New Mexico, Pennsylvania, Texas, and Washington each reported a single case.

^†^ Percentages might not sum to 100 because of rounding.

^§^ Laboratory-confirmed infection with *Salmonella* Typhimurium (allele code SALM1.0 – 6745.4.2.1x) that was determined by whole-genome sequencing to be closely related to other isolates in the outbreak in a person with symptom onset during September 15, 2023–May 4, 2024.

^¶^ Laboratory-confirmed *Salmonella* infection without serotype and whole-genome sequencing results in a person reporting brand A raw milk consumption during the same symptom onset date range as that of confirmed cases.

** Includes confirmed and probable cases.

^††^ Includes milk and at least one of the following raw dairy products: cheese, heavy cream, or kefir.

### Reported Raw Dairy Product Exposure

Information on raw dairy product exposure was available from case report forms or supplementary questionnaires for 91 (53%) patients; among these, 79 (87%) and 12 (13%) had illnesses that met the confirmed and probable case definitions, respectively. The remaining 80 (47%) patients with confirmed salmonellosis either could not recall their exposure history or were lost to follow-up. Among the 91 interviewed patients, 72 (79%) reported consuming liquid raw milk products, including raw milk (71) and raw heavy cream (one); 67 (93%) of these patients reported consuming brand A raw milk or raw heavy cream during the 7 days preceding illness onset. The remaining 19 (21%) reported that they did not consume liquid raw milk. In addition, one patient reported consuming brand A raw milk cheese only. Among the 79 patients with confirmed cases, 55 (70%) reported exposure to brand A raw milk or heavy cream; this percentage was significantly higher than the expected background rate of raw milk consumption in the general California population (1.9%; p<0.001), based on the 2018–2019 FoodNet Population Survey. Because patients with probable cases consumed brand A raw milk by definition, these persons were excluded from the statistical analysis.

### Environmental Health Investigation

On October 19, 2023, CDPH and CDFA notified dairy farm A of the initial nine cases of salmonellosis associated with brand A raw milk, including the eight San Diego County cases and one from another LHD. During October 2023, CDPH, CDFA, and LHDs collected 40 brand A products (raw milk, cheese, heavy cream, and kefir) from the dairy farm, retail stores, and patients’ homes. Samples were tested for *Salmonella* and underwent WGS if *Salmonella* test results were positive. CDPH initiated a traceback investigation of brand A raw milk to identify product lot numbers and production dates associated with contaminated products. On October 23, 2023, CDPH and CDFA notified dairy farm A that *S*. Typhimurium was detected in a sample from brand A bottled raw milk collected from the farm on October 19.

*S.* Typhimurium was detected in three of the 40 brand A product samples collected; isolates were indistinguishable by WGS from the clinical isolates collected from patients in the outbreak. The positive samples included two from bottled raw whole milk collected by CDFA at the bottling facility operated by dairy farm A on October 19 and 25, 2023; one retail sample of raw milk with a best-by date of October 27, 2023. No *Salmonella* bacteria were detected in the remaining samples from retail stores and patients’ homes. In addition, the outbreak strain was detected in a sample of raw cheese aged for 60 days made from the contaminated milk and collected from the dairy during January 2024; *S*. Typhimurium was not detected in any raw milk cheese samples collected before January 2024.

## Public Health Response

On October 24, 2023, in response to the epidemiologic evidence and the *Salmonella-*positive raw milk sample, dairy farm A halted production and voluntarily recalled its raw milk. The recall included fluid milk and heavy cream with best-by dates of October 11–November 6, 2023; recalled lots were destroyed or held at the facility for aged cheese production, with cheese to be held under impound by CFDA. Raw cheese made from the contaminated milk lots tested positive after 60 days of aging and was not distributed for retail sale. CDFA conducted a sanitary inspection of the raw milk bottling and cheese making plant on October 25, and a joint inspection of the dairy farm on October 27 along with county environmental health inspectors. The farm’s internal testing detected *Salmonella* in milk from a recently purchased cow; the isolate was not further characterized. The cow was removed from the herd, and subsequent testing of the herd did not detect *Salmonella*. These efforts met CDFA requirements, allowing production to recommence on October 31, 2023. Extensive public messaging regarding this outbreak was issued by LHDs and CDPH in October 2023, including press releases and social media posts. Messaging instructed consumers to discard any recalled brand A milk or heavy cream; advised those experiencing symptoms of salmonellosis, including diarrhea, fever, vomiting, or abdominal pain to seek medical care; and recommended consuming pasteurized dairy products to prevent foodborne illnesses.

## Discussion

Consumption of commercially distributed raw milk resulted in a large and widespread salmonellosis outbreak that disproportionately affected young children. The median age of reported ill patients was 7 years, and children were those most likely to be hospitalized among all age groups.

This outbreak is one of the largest foodborne outbreaks linked to raw milk in recent U.S. history. During 2009–2021, a total of 143 enteric disease outbreaks that were confirmed or suspected to be associated with consumption of raw milk were reported to CDC (*6*). Of these, 16 were salmonellosis outbreaks, all of which were small (median number of cases = 10; range = 2–33). In California, four confirmed and three suspected enteric disease outbreaks have been linked to raw milk since 2012; no outbreaks associated with pasteurized milk have occurred during this period (CDPH, Infectious Diseases Branch, Disease Investigations Section, unpublished data, 2018–2021). In addition, this investigation confirmed that cheese produced from raw milk, even if aged for 60 days, has the potential to remain contaminated with *Salmonella*. The Food and Drug Administration permits the distribution of hard cheeses made from raw milk if they are aged for ≥60 days (*7*).

Rapid, accurate recognition of the likely outbreak source by an LHD and close collaboration between local and state health agencies resulted in an expedited and focused investigation and timely product recall; time from initiation of CDPH investigation to product recall was 1 week. Enhanced surveillance sampling by CDFA and CDPH and WGS of milk and clinical isolates were critical to confirming the source of the outbreak and facilitating the recall.

The source of the illness in the four non-California residents is unknown. Federal law prohibits the sale of raw milk for human consumption across state lines, and none of these four patients reported travel to California. However, federal law does not prohibit the interstate sale of raw milk intended for pet consumption or interstate sale of raw cheese aged for ≥60 days. One of the four non-California patients reported purchasing dairy farm A raw milk (and possibly cheese) in their state of residence, the second patient consumed dairy farm A raw cheese only from an unknown purchase location, the third patient did not recall consuming any raw dairy products, and the fourth patient was lost to follow-up. These patients could have purchased raw milk for pets or raw cheese within their states. Other possible explanations for the non-California outbreak cases are that patients could not recall or did not accurately report travel history or were infected through unrecognized secondary transmission.

### Limitations

The findings in this report are subject to at least three limitations. First, cases continued to occur after the product was recalled, and not all patients with confirmed cases reported consuming raw milk. In any outbreak, some cases linked to the outbreak by laboratory data have uncertain exposure routes; intermittent cases might have resulted from indirect spread, such as through person-to-person transmission. Patients might also have had the products in their homes and continued to use them after the recall. Second, the interval between a patient’s illness onset and reinterview with the supplementary questionnaire might have resulted in poorer recall of exposure history than that during the initial interview, introducing recall bias. Finally, patients might have chosen to not disclose their raw dairy product consumption history.

### Implications for Public Health Practice

Consumption of raw dairy products, even from a licensed producer, continues to present a risk for enteric and other infectious diseases, especially among children. Public health agencies should continue to emphasize this message to help prevent future foodborne disease outbreaks, with educational efforts focusing on populations at high risk for complications from infection, including children (through their parents), as well as pregnant women and persons with immunocompromise (*8*). Systems for collaboration among local, state, and federal public health and regulatory partners and surveillance testing should be supported and enhanced to facilitate rapid evidence-based action during future foodborne outbreaks.

## Footnotes

* Title 17 Revisions, Section 2500 of the California Code of Regulations | CDPH; Salmonellosis | Information for Local Health Departments | CDPH

† Confirmed outbreak strain was *S.* Typhimurium with allele code SALM1.0 – 6745.4.2.1x and highly related (within two alleles) using core genome multilocus sequence typing or on the single nucleotide polymorphism (SNP) branch (0–6 SNPs) using the National Center for Biotechnology Information SNP analysis. Pathogen Detection | National Library of Medicine

§
About PulseNet | PulseNet | CDC
